# Glucagon-Like Peptide 1: A Predictor of Type 2 Diabetes?

**DOI:** 10.1155/2017/7583506

**Published:** 2017-09-10

**Authors:** Matthias Ploug Larsen, Signe Sørensen Torekov

**Affiliations:** Department of Biomedical Sciences and Novo Nordisk Foundation Center for Basic Metabolic Research, Faculty of Health Sciences, University of Copenhagen, Copenhagen N, Denmark

## Abstract

**Background:**

The incretin effect is impaired in patients with type 2 diabetes.

**Aim:**

To assess the relation between the incretin hormone GLP-1 and the prediabetic subtypes: impaired fasting glucose (IFG), impaired glucose tolerance (IGT), and the combined IFG/IGT to investigate whether a low GLP-1 response may be a predictor of prediabetes in adults.

**Method:**

298 articles were found using a broad search phrase on the PubMed database and after the assessment of titles and abstracts 19 articles were included.

**Results and Discussion:**

Studies assessing i-IFG/IFG and i-IGT/IGT found both increased, unaltered, and reduced GLP-1 levels. Studies assessing IFG/IGT found unaltered or reduced GLP-1 levels. When assessing the five studies with the largest sample size, it clearly suggests a decreased GLP-1 response in IFG/IGT subjects. Several other factors (BMI, glucagon, age, and nonesterified fatty acids (NEFA)), including medications (metformin), may also influence the secretion of GLP-1.

**Conclusion:**

This review suggests that the GLP-1 response is a variable in prediabetes possibly due to a varying GLP-1-secreting profile during the development and progression of type 2 diabetes or difference in the measurement technique. Longitudinal prospective studies are needed to assess whether a reduced GLP-1 response is a predictor of diabetes.

## 1. Introduction

The number of people diagnosed with diabetes globally was increased from 108 million adults in 1980 to an estimated 422 million in 2014 [1]. Thus, the global prevalence of diabetes has increased from 4,7% to 8,5% from 1980 to 2014 [1]. Diabetes causes complications with a 2-3 times higher rate of cardiovascular disease, a 10–20 times higher rate of lower extremity amputations, and a 10 times higher incidence of end-stage renal disease in diabetic adults compared to nondiabetic adults [1]. Along with this, diabetic retinopathy caused 2,6% of total blindness and 1,9% of total moderate or severe visual impairment globally in 2010 [1]. In addition to these complications, diabetes entails economic costs for both the individual and the health systems [1] and the global cost of diabetes for 2015 was estimated to be US$1,3 trillion corresponding to 1,8% of global gross domestic product (GDP) [2]. Further knowledge about aetiology and pathogenesis could contribute to turn over this development.

When glucose is orally ingested, it elicits a much greater insulin response (two- to threefold) than if glucose is intravenously injected to give the same blood glucose level. This phenomenon is called the incretin effect and is due to the secretion of glucagon-like peptide 1 (GLP-1) and glucose-dependent insulinotropic peptide (GIP) which increases the glucose-induced insulin secretion [3]. In patients with type 2 diabetes, the incretin effect is impaired [4]. In addition to that, studies concerning GLP-1 secretion in patients with type 2 diabetes during an oral glucose tolerance test (OGTT) have observed both unaltered [5, 6] and reduced [7] GLP-1 responses, suggesting a varying GLP-1-secreting profile during the development and progression of type 2 diabetes [5, 7] or difference in the measurement technique [8]. This is further supported by the observation that several factors (BMI, glucagon, age, and nonesterified fatty acids (NEFA)), including medications (metformin), influence the secretion of GLP-1 [6].

The biggest study to date—a large Danish study—published in 2015, involving 1462 individuals, demonstrated a reduced GLP-1 response to an OGTT in prediabetes, screen-detected type 2 diabetes, and obese and overweight individuals compared to normal glucose-tolerant individuals and normal weight individuals, respectively [9]. Individuals diagnosed with prediabetes are at an increased risk of developing type 2 diabetes, with a yearly progression rate of 3,5%–7,0% in individuals with prediabetes compared to a yearly progression rate of 2% in normoglycemic individuals [10]. In continuation to that, a reversing of the prediabetic state towards normal glucose regulation is associated with an up to 56% lower risk of developing type 2 diabetes compared to individuals remaining prediabetic [10]. Further knowledge about aetiology and pathophysiology of prediabetes and the progression to type 2 diabetes might help in preventing and treating both states [9, 11]. Additionally, more extensive knowledge could help improve the diagnostic criteria concerning the subtypes of prediabetes [11].

Therefore, this review will assess the relation between the incretin hormone GLP-1 and the prediabetic state and investigate whether GLP-1 may be a predictor of prediabetes in adults.

## 2. Research Question

Is a low GLP-1 response a predictor of prediabetes in adults?

## 3. Materials and Methods

This review is a literature study using the electronical PubMed database to find relevant literature. Only articles in English were included. The following search phrase in PubMed was used:

(GLP-1) AND (prediabet∗OR pre-diabet∗OR “impaired glucose tolerance” OR “impaired glucose tolerant” OR IGT OR “impaired fasting glucose” OR “impaired fasting glycaemia” OR IFG OR intermediate hyperglycaemia).

The last search update was conducted on 10 March 2017.

Articles were included which investigated the relationship between GLP-1 and the different subtypes of prediabetes: impaired fasting glucose (IFG), impaired glucose tolerance (IGT), and the combined IFG/IGT.

The search identified 298 articles. These 298 articles were first evaluated based on their title, and irrelevant articles, in relation to the inclusion criteria and articles in languages other than English, were excluded. Articles with inadequate or indefinite titles were included for further evaluation. Articles being irrelevant in proportion to answering the research question was excluded. For example, articles investigating the impact of surgical or pharmacological interactions on the GLP-1 response and articles not involving prediabetic subjects. Articles assessing GLP-1 alterations in adolescents were also excluded.

**Figure 1:**
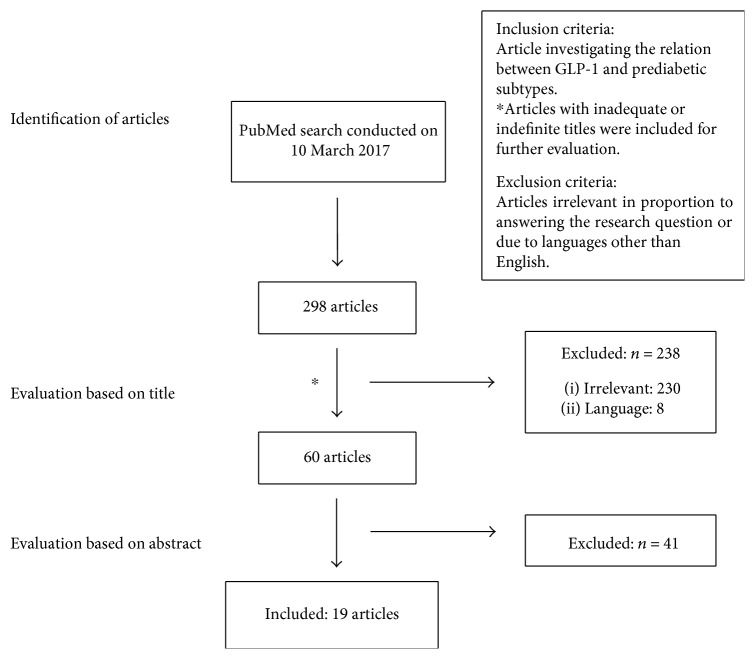
Flowchart of the search process. See text for details.

Finally, this resulted in 60 articles. These 60 articles were evaluated based on their abstracts, and relevant articles fulfilling the inclusion criteria were read and included in the review. Included articles in the review are 19. A flowchart of the search process is illustrated in Figure 1.

## 4. GLP-1 and Prediabetes: Is There a Connection?

### 4.1. GLP-1—An Incretin Hormone

The incretin effect is mediated by the incretin hormones—the two major being GLP-1 and GIP. GLP-1 is a gut peptide secreted from open-type enteroendocrine L cells—located in the intestinal mucosa—in response to ingested nutrients (carbohydrate, protein, and fat). This is thought to be the primary stimulus for secretion, and the secretory response depends on both meal size and gastric emptying rate. Neuronal and hormonal mechanisms have also been proposed regarding the regulation of secretion [3].

**Figure 2:**
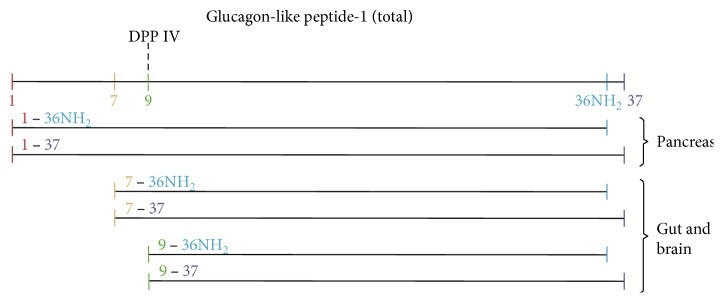
GLP-1 isoforms—illustration from Bak et al. [8].

GLP-1 comes in different isoforms (see Figure 2): unamidated GLP-1(1–37), GLP-1(7–37), and GLP-1(9–37), and amidated GLP-1(1–36)NH_2_, GLP-1(7–36)NH_2_, and GLP-1(9–36)NH_2_ [8]. In human, the amidated isoforms are the predominant [8, 12]. GLP-1(7–36)NH_2_ and GLP-1(7–37) are termed “active/intact” GLP-1 and when secreted, both configurations of the hormone are rapidly degraded (*T*_1/2_ = 1-2 min) by the enzyme dipeptidyl peptidase 4 (DPP-4) to GLP-1(9–36)NH_2_ and GLP-1(9–37), respectively, leaving only around 10–15% of the “active/intact” GLP-1 in the systemic circulation [3, 5, 12, 13]. Furthermore, GLP-1 is metabolised by the enzyme neutral endopeptidase 24.11. [12, 13]. GLP-1(7–36)NH_2_, GLP-1(7–37), and the metabolites GLP-1(9–36)NH_2_ and GLP-1(9–37) are termed “total” GLP-1 [12, 13]. “Active/intact” GLP-1 only accounts for the endocrine effect of GLP-1, whereas GLP-1 is also thought to have neural effects, whereby measuring “total” GLP-1 mirror the total effect of GLP-1 better [12]. Furthermore, since the concentration of “active/intact” GLP-1 is very low (0–15 pmol/l) and rises only very little in response to small meals, it is harder to detect a difference in secretion compared to the measuring of “total” GLP-1 ranging from 5 to 80 pmol/l [5, 8, 12, 13]. Therefore, it has been argued that measuring “total” GLP-1 is best, when GLP-1 secretion should be measured [5, 8, 12–14]. In *α* cells, processing of the proglucagon (PG) gene leads to the secretion of small amounts of the biologically inactive peptides GLP-1(1–36)NH_2_ and GLP-1(1–37) [12]. Due to cross-reactivity and the varying commercially available assays, knowledge of this plethora of peptides and their metabolites is important, when assessing the assays used to measure their plasma concentrations [8, 12].

Besides increasing insulin secretion, GLP-1 also has other effects, including inhibition of glucagon secretion from *α* cells; stimulating, potential proliferating, and antiapoptotic effects on *β* cells; a delay of gastrointestinal secretion and motility; an appetite-reducing effect; a potential advantageous effect on the cardiovascular system; and a potential neurotropic or neuroprotective effect [3, 13]. Given that a lot of these effects are advantageous in the scope of treating diabetes, the use of incretin-based therapies is increasing [3].

### 4.2. Prediabetes—Not Just One Condition

Prediabetes is an overall term to describe the dysglycemic conditions between normal glucose tolerance (NGT) and the diabetic state. The different subtypes of prediabetes consist of isolated impaired fasting glucose (IFG), isolated impaired glucose tolerance (IGT), and the combined state IFG/IGT [10, 11]. An estimation of the worldwide prevalence of individuals with prediabetes is approaching 840 million [10]. Since people with prediabetes are at an increased risk of developing diabetes compared to non-prediabetic individuals—as mentioned above—screening for the prediabetic state, and thereby initiating prevention of development of diabetes, is a health goal [10]. This is further supported by studies showing an increased risk of micro- and macrovascular complications with prediabetes, exemplified by a study showing that 8% of nondiabetic participants in a cross-sectional analysis had diabetic retinopathy, and an increased risk of cardiovascular disease at ≈20% in prediabetes [10]. It is hypothesised that the different prediabetic states have both different aetiology and pathophysiology and that individualised prevention and treatment strategies should be assessed based on the prediabetic subtype [10, 11]. Different diagnostic criteria with varying cut-points exist and have changed through time [1, 10, 15, 16].

### 4.3. The Relation between GLP-1 and Prediabetes

Based on the literature search in PubMed, 19 original articles are included in assessing the relation between GLP-1 and prediabetes. In the following, these articles will be reviewed.

The included studies were published between 1997 and 2016. Not all studies investigated all the different subtypes of prediabetes described above. For clarity, and since it appears that the different subtypes have both different aetiology and pathophysiology [10, 11], the results will be reviewed in order of subtype. The included studies are not consistent in the terms used to describe the prediabetic subtype (i-IFG or IFG and i-IGT or IGT). The term used when assessing an included study is the one used in the respective study. An overview of the results can be seen in Table 1.

#### 4.3.1. Stimulus

All studies, except two [17, 18], conducted a 75 g OGTT measuring GLP-1, and other variables, at various time points. Fernandez-Garcia et al. [17] conducted a 60 g high-fat meal and Toft-Nielsen et al. [18] conducted a mixed breakfast meal containing 2250 kJ.

Besides the 75 g OGTT, six studies [19–24] conducted additional tests on the same participants: Yabe et al. [19] and Lee et al. [20] conducted a meal tolerance test (MTT) (480 kcal, carbohydrate : protein : fat = 2.8 : 1 : 1). Vollmer et al. [24] conducted a mixed meal challenge (820 kcal, carbohydrate : protein : fat = 3.38 : 1 : 3.30). Faerch et al. [21] and Laakso et al. [23] conducted an intravenous glucose tolerance test (IVGTT) to measure first-phase insulin secretion and a 120 min hyperinsulinaemic-euglycaemic clamp to measure peripheral insulin sensitivity. Muscelli et al. [22] conducted an isoglycaemic intravenous glucose test to assess the incretin effect.

#### 4.3.2. “Active/Intact” or “Total” GLP-1

The included studies have measured both “active/intact” and “total” GLP-1 (and eventually the small amount of GLP-1(1–36)NH_2_ and GLP-1(1–37) although not mentioned in any of the studies).

Four studies measured “active/intact” GLP-1 only [17, 20, 25, 26], thirteen studies measured “total” GLP-1 only [7, 9, 11, 18, 22–24, 27–32], and two studies measured both “active/intact” and “total” GLP-1 [14, 19]. As mentioned earlier and discussed later, measuring “total” GLP-1 is best, when GLP-1 secretion should be measured [5, 8, 12–14]. The studies measuring “active/intact” GLP-1 only will not be reviewed in detail, since they are concluding on metabolites which they may not have measured. Results assessing “active/intact” GLP-1 can be seen in Table 1. Additionally, when measuring “active” GLP-1, it is advised to use a DPP-4 inhibitor when collecting blood samples [12]. This was done in all studies except Fernandez-Garcia et al. [17] which did not report any use of DPP-4 inhibitor. This could of course have influenced the results.

#### 4.3.3. “Total” GLP-1

*(1) “Total” GLP-1 and i-IFG/IFG*. 6/19 included studies assessed the relation between “total” GLP-1 and i-IFG/IFG [9, 14, 21, 23, 27, 29].

Increased: Faerch et al. [21] studied the GLP-1 response to a 3 h OGTT in 66 subjects. They found no difference in fasting GLP-1 between i-IFG and NGT. However, they found a significantly higher 3-hour AUC GLP-1 in i-IFG subjects compared to NGT subjects. There was a low number of women in this group (only 2). This could suggest a compensatory GLP-1 response in this subgroup.

Unchanged: Hussein et al. [27] studied 80 subjects' glucose-stimulated GLP-1 response (30 minutes after 75 g glucose). The subjects were divided into 4 groups: normal weight NGT, obese NGT, obese IFG, and obese IFG/IGT. They found no difference in glucose-stimulated GLP-1 between obese NGT and obese IFG. However, glucose-stimulated GLP-1 were reduced in all obese groups compared to the normal weight group. These results indicate that BMI, not glucose tolerance, influences 30 min-glucose-stimulated GLP-1 levels.

Zhang et al. [29] studied 531 subjects' GLP-1 response to a 2 h OGTT. They only measured GLP-1 at 0 and 120 min and found no difference in fasting GLP-1, 2 h GLP-1, or ΔGLP-1 when comparing i-IFG and NGT subjects.

Smushkin et al. [14] studied 165 subjects' GLP-1 response to a 2 h OGTT. They found no difference in either fasting GLP-1, max “total” GLP-1, AUC GLP-1, or AAB GLP-1 when comparing IFG/NGT and NFG/NGT subjects.

Reduced: Faerch et al. [9] studied 1462 subjects' response to a 2 h OGTT. They measured GLP-1 at 0, 30, and 120 min and grouped the subjects in proportion to sex. They found no differences in the male i-IFG compared to NGT. However, in the female groups, they found a reduced rAUC_0–30_, rAUC_0–120_, and iAUC_120_ when comparing i-IFG and NGT subjects. This suggests that the GLP-1 response is influenced by sex.

Laakso et al. [23] studied 278 subjects' GLP-1 response to a prolonged 4 h OGTT. All subjects were nondiabetic offspring of patients with type two diabetes. They found a reduced GLP-1 response at 15, 90, and 120 min and a reduced AUC_0–240_ when comparing i-IFG and NGT subjects. This could indicate that being a nondiabetic offspring of a patient with type 2 diabetes could influence the GLP-1 response suggesting a genetic component in the GLP-1 response.

*(2) “Total” GLP-1 and i-IGT/IGT*. 14/19 included studies assessed the relation between “total” GLP-1 and i-IGT/IGT [7, 9, 14, 18, 19, 21–24, 28–32].

Increased: Smushkin et al. [14] also studied the GLP-1 response between NFG/IGT and NFG/NGT. They found no difference in fasting GLP-1, max GLP-1, and AUC GLP-1. However, they found an increased integrated incremental concentration of GLP-1 (2-hour area above basal (AAB GLP-1)) when comparing NFG/IGT and NFG/NGT.

Unchanged: Wang et al. [7] studied 80 subjects' GLP-1 response to a 3 h OGTT. They found no difference in either GLP-1 levels at each measured time point, ΔGLP-1, or 3-hour AUC GLP-1 when comparing IGT and NGT subjects.

Yabe et al. [19] conducted both a 2 h OGTT and a MTT in 102 subjects. They found no difference in either fasting nor postprandial GLP-1 response—both when assessing GLP-1 levels at each time point and 2 hour AUC GLP-1—between IGT and NGT.

Faerch et al. [21] found no difference in either fasting GLP-1 or 3-hour AUC GLP-1 when comparing i-IGT and NGT subjects.

Muscelli et al. [22] studied 51 well-matched subjects' GLP-1 response to a 3 h OGTT. They found no difference in GLP-1 levels or 3-hour AUC GLP-1 between IGT and NGT subjects.

Vollmer et al. [24] studied 48 well-matched subjects' GLP-1 response to a 4-hour OGTT and a mixed meal. They found no difference in GLP-1 levels when comparing IGT and NGT in both challenges.

Rask et al. [31] studied 30 well-matched women in a 3-hour OGTT and measured their GLP-1 response. They found no difference in fasting GLP-1, GLP-1 levels at each time point, 30 min AUC GLP-1, and 120 min AUC GLP-1 between IGT and NGT. However, they found a significant difference when comparing the “first 30 minutes GLP-1 concentration increase'—the increase being reduced in IGT versus NGT. The 30 min iAUC GLP-1 also showed a tendency (*P* = 0,072) to be reduced in IGT versus NGT. 17 of the women were postmenopausal, which could have an impact on the results.

Toft-Nielsen et al. [18] studied 102 subjects' GLP-1 response to a 4-hour mixed meal. They found no difference in either fasting GLP-1 or 4-hour AUC GLP-1 (corrected for BMI and gender) between IGT and NGT subjects.

Ahren et al. [32] studied 13 well-matched postmenopausal women and measured their GLP-1 response in a 2-hour OGTT. They found no difference in fasting GLP-1, no difference in GLP-1 increase AUC_0–60_, or GLP-1 decrease between IGT and NGT.

Reduced: Faerch et al. [9] also measured GLP-1 in i-IGT subjects. They found a reduced rAUC_0–30_ and a reduced rAUC_0–60_ in women with i-IGT compared to NGT. No differences were found between the two groups when assessing iAUC_30_ or iAUC_60_.

Shen et al. [28] studied 43 subjects' GLP-1 response to a 2-hour OGTT. They found no difference in fasting GLP-1. However, GLP-1 levels were reduced at 30, 60, and 90 min and 120 min AUC GLP-1 was reduced when comparing IGT and NGT.

Zhang et al. [29], mentioned above in the i-IFG/IFG part, found no differences in fasting GLP-1 or 2 h GLP-1 between subjects with i-IGT and NGT. However, they found that ΔGLP-1 was reduced in i-IGT versus NGT.

Nathanson et al. [30] studied 509 71-year-old men and their GLP-1 response to an OGTT measuring GLP-1 at 0 and 60 minutes. They found no reduction in fasting GLP-1 and GLP-1 at 60 min. However, ΔGLP-1 was reduced in IGT compared to NGT subjects.

Laakso et al. [23], also mentioned above, found reduced GLP-1 levels at 15, 90, and 120 minutes and a reduced AUC_0–240_ in i-IGT versus NGT.

*(3) “Total” GLP-1 and IFG/IGT*. 5/19 included studies assessed the relation between “total” GLP-1 and IFG/IGT [9, 14, 23, 27, 29].

Unchanged: Hussein et al. [27] also assessed IFG/IGT subjects. They found no difference in the GLP-1 response in obese IFG/IGT subjects compared to obese NGT.

Smushkin et al. [14] found no difference in either fasting or postprandial GLP-1 when comparing both maximal GLP-1, 2-hour AUC and 2-hour AAB, in IFG/IGT with NFG/NGT.

Reduced: Faerch et al. [9], as mentioned above, stratified their subjects according to sex. When comparing IFG/IGT with NGT subjects, they found a reduced 120 min GLP-1 in both sexes. Furthermore, they found a reduction in rAUC_0–30_, rAUC_0–120_, and iAUC_120_ in female subjects with IFG/IGT.

Zhang et al. [29] found a reduced fasting GLP-1 when comparing IFG/IGT with i-IGT. When comparing IFG/IGT with both NGT, i-IFG and i-IGT, they found a reduced 2-hour GLP-1 and a reduced ΔGLP-1.

Laakso et al. [23] found a reduced 15, 90, and 120 min GLP-1 and a reduced AUC_0–240_ when comparing IFG/IGT with NGT subjects.

### 4.4. Why Such a Difference?

Summarizing, studies assessing the “total” GLP-1 (and “active/intact”) response in i-IFG/IFG and i-IGT/IGT found both increased, unaltered, and reduced GLP-1 levels when compared with NGT. Studies assessing IFG/IGT found unaltered or reduced GLP-1 levels when compared with NGT. Thereby, nearly all possible outcomes have been reported. So why this difference?

#### 4.4.1. “Active/Intact” or “Total” GLP-1

As mentioned earlier, it is argued that “total” GLP-1 is best suited when GLP-1 secretion should be assessed [5, 8, 12–14]. The choice of measuring “active/intact” GLP-1 could be one reason of conflicting results. Furthermore, when assessing “active/intact” GLP-1, a difference in DPP-4 activity could also have an impact on the results—although included studies show no difference in DPP-4 activity among prediabetic subgroups [14, 19, 20, 26].

#### 4.4.2. Study Design and Duration

All the included studies were cross-sectional studies except the study by Zheng et al. [25]. This type of study design is not designed to assess the duration by which individuals have had prediabetes [21, 33]. This could have influenced the results, and longitudinal prospective studies are therefore suggested to assess the GLP-1 response in the course from NGT over prediabetes to type 2 diabetes [6, 11, 21, 34]. Only two studies have an estimate of prediabetes duration: Zheng et al. found a reduced fasting “active/intact” GLP-1 in prediabetic subjects that were NGT 4 years earlier, and Faerch et al. [21] studied individuals that were NGT 5 years earlier finding no difference in fasting “total” GLP-1 but found an increased 3-hour AUC GLP-1 in i-IFG versus NGT. This could suggest a compensatory GLP-1 secretion [21], which could perhaps explain the increased 2 h AAB “total” GLP-1 response in NFG/IGT versus NFG/NGT subjects, reported by Smushkin et al. [14].

#### 4.4.3. Diagnostic Criteria

As mentioned earlier, different diagnostic criteria with different cut-points for the prediabetic subtypes exist and have varied through time [1, 10, 15, 16]. The current definition of IGT from WHO corresponds to the term IFG/IGT used by many studies [1]. However, many of the included studies include cut-points to differentiate between i-IFG, i-IGT, and IFG/IGT although varying terms are used (i-IFG or IFG and i-IGT or IGT) [9, 11, 14, 17, 19, 23, 27–29]. This could also be a reason for the differing results. Furthermore, it has been argued that i-IFG and i-IGT are a continuum of impaired glucose regulation rather than absolute states [11], hence also affecting the results due to defined diagnostic criteria.

#### 4.4.4. Stimulus

Most of the studies conducted an OGTT, but three studies conducted an additional meal test, as described above [19, 20, 24]. The three studies found the same results in both challenges, suggesting that stimulus is not influencing on the different results in the included studies. Vollmer et al. [24] found no significant difference in integrated incremental plasma GLP-1 concentrations when comparing OGTT with a mixed breakfast meal. Furthermore, peak GLP-1 were seen after 30 minutes during the OGTT and after 90 minutes during the mixed meal. Yabe et al. [19] reported that “total” GLP-1 increased only after glucose ingestion and not after a mixed meal. In contrast to Vollmer et al., Lee et al. [20] reported a peak of “active/intact” GLP-1 at 20–30 minutes in both challenges, concentrations after the OGTT: 7–9 pmol/l and the MTT: 3–5 pmol/l, and a significantly greater iAUC GLP-1 in OGTT versus MTT. This difference could be explained by the different meal compositions or measuring technique.

#### 4.4.5. Study Size

The study size in the included studies varied from 13 to 1462. A lot of the studies are therefore limited by their sample size. When assessing the five studies with the largest sample sizes (>200 subjects) [9, 23, 25, 29, 30], it suggests a reduced GLP-1 response (for details, see Table 1) in IFG/IGT. Altering results are reported in the i-IFG/IFG and i-IGT/IGT groups due to, for example, sample times, differing analytical methods, and sex. This strongly suggests an alteration in the GLP-1 response in the IFG/IGT group.

#### 4.4.6. Assays

Differences in assays may have influenced the results, both in relation to epitope (e.g., “active/intact” or “total” GLP-1) and selected commercially available kits [8, 12]. Regarding epitope, Smushkin et al. [14] used a different approach to measure “total” GLP-1 by measuring active and inactive GLP-1 and add the two to get the “total” GLP-1. By choosing that approach, they might have avoided the detection of the small amounts of GLP-1(1–36)NH_2_/(1–37) secreted from the pancreas, which all C-terminally specific antibodies might detect [12]. The plethora of selected kits in different studies might also have influenced the results [8]. Interestingly, Zhang et al. [29] used a kit, which Bak et al. [8] found not to detect any GLP-1 isoforms in both plasma or buffer.

#### 4.4.7. Sampling Time

Sampling time might also be a reason for the difference in reported results. Both the OGTT duration and the sampling interval varied considerable between studies. With large intervals, the peak GLP-1 might be missed emphasizing frequent sampling in future studies [34]. Peak GLP-1 could vary with varying glucose tolerance status [14] or gastric emptying [34].

#### 4.4.8. Sex

Faerch et al. [9] found a higher 30 and 120 min, tAUC, and rAUC GLP-1 response in women compared to men, when adjusting for BMI, height, and weight. This is supported by Vollmer et al. [24] reporting higher GLP-1 plasma concentrations in women compared to men in both an OGTT and a mixed meal and by Toft-Nielsen et al. [18] reporting a reduced AUC GLP-1 in males. If not corrected, this could also be a reason of the different results.

#### 4.4.9. BMI

Faerch et al. [9] also found a relation between BMI and the GLP-1 response, reporting a reduced rAUC_0–30_, rAUC_0–120_, iAUC_0–30_, and iAUC_0–120_ in both overweight and obese compared to normal weight individuals, and adjusted for glucose tolerance status, age, and sex. This is supported by several other included studies [18, 22, 24, 27]. Not all studies had matched BMI between the groups. If not corrected, this could also be a reason of the different results.

#### 4.4.10. Genetics

Laakso et al. [23] found a reduced GLP-1 response in i-IFG, i-IGT, and IFG/IGT. All participants were nondiabetic offspring of patients with type 2 diabetes. This could indicate a genetic component in the GLP-1 response and the development of prediabetes and could further be a reason of the differing results between the included studies.

#### 4.4.11. Ethnicity

The included studies are conducted in different ethnic groups. This could also have an impact on the differing results [21, 35].

#### 4.4.12. Analytical Methods

Different analytical methods can also have influenced the results between the studies [9]. Faerch et al. [9], for example, used rAUC, not used in any of the other studies.

#### 4.4.13. Age

Faerch et al. [9] also reported a relation between GLP-1 and age, with an increasing—although small—GLP-1 response with increasing age. If not corrected, this could also explain the differing results.

#### 4.4.14. Other Factors

Other factors could also have an influence on the GLP-1 response, for example, insulin resistance [17, 36], glucagon levels [6], and nonesterified fatty acid levels [6, 24].

### 4.5. Potential Mechanisms for the Eventually Reduced GLP-1 Response in Prediabetes

Rask et al. [36] have found a reduced GLP-1 secretion in response to a mixed meal in nondiabetic men with insulin resistance. This suggests an association between insulin resistance and GLP-1 secretion. *In vitro* studies of models of L cells have shown that L cells express the insulin receptor [37]. Furthermore, *in vitro* studies have shown a stimulatory effect of insulin on the GLP-1 secretion in L cell models in a glucose-dependent manner [37, 38]. In continuation of the study by Rask et al. [36], *in vitro* and *in vivo* studies have showed reduced homologous and heterologous secretagogue-induced GLP-1 secretion in insulin resistant L cell models [37]. A study by Iepsen et al. [39] showed an increase in the meal-induced secretion of GLP-1 after a 1-year 13% body weight loss maintenance accompanied by a significant improvement in the HOMA-IR [39] further indicating that the L cell might be insulin sensitive [9, 39]. This potential mechanism could differ between the prediabetic subtypes since the site of insulin resistance is hypothesised to be different with increased hepatic glucose production in IFG and a reduced peripheral glucose disposal in IGT [10, 11].

*In vitro* studies have also shown a glucotoxic effect on the GLUTag L cell model reducing acute glucose-induced GLP-1 secretion [40] and a lipotoxic effect on the GLUTag L cell model affecting L cell viability, with a presumed counteractive effect of insulin and the GLP-1 analog Exendin-4 [38].

Furthermore, *in vitro* studies have shown that chronic exposure to the proinflammatory cytokine TNF*α* reduces both GLP-1 expression and secretion from L cell models expressing the TNF*α*-receptor TNFR1 [41]. This could indicate a role of these extracellular metabolites and TNF*α* on the potentially reduced GLP-1 secretory response in prediabetes.

Returning to the research question, Is a low GLP-1 response a predictor of prediabetes in adults? When assessing the five studies with the largest sample size, it clearly suggests an alteration in the GLP-1 response in IFG/IGT subjects and varying results when assessing the two other subtypes—i-IFG/IFG and i-IGT/IGT. However, varying results have been reported in all subtypes and warrants further studies. Possible reasons for the varying results have been discussed. As mentioned above, the need for longitudinal prospective studies are necessary to assess the impact of duration of the prediabetic state on the GLP-1 response in prediabetes and to determine the, eventual, temporal influence of the GLP-1 response in the pathogenesis of type 2 diabetes. Additionally, the aim of this review is to survey the studies assessing the relation between the GLP-1 response and the different prediabetic subtypes and to suggest potential confounders relevant when conducting future studies. An eventual limitation of this review is that only the PubMed database was assessed. Furthermore, relevant studies could have been excluded in the search or in the evaluation of relevant studies. To avoid the exclusion of studies, a broad search was conducted including many synonyms for the rather broad term “prediabetes.”

## 5. Conclusion

Conclusively, this review suggests that the GLP-1 response is a variable in prediabetes possibly due to a varying GLP-1-secreting profile during the development and progression of type 2 diabetes or difference in the measurement technique. Longitudinal prospective studies are needed to assess whether a reduced GLP-1 response is a predictor of diabetes. Furthermore, this review gives an overview of studies assessing the relation between GLP-1 and prediabetes and discusses possible confounding factors, relevant when conducting future studies.

## Figures and Tables

**Table 1:** Overview of results.

Author and year	Study size (*n*)	Stimulus	Total or active/intact GLP-1 and assay/detection method	GLP-1 levels: i-IFG/IFG	GLP-1 levels: i-IGT/IGT	GLP-1 levels: IFG/IGT	BMI	Age	Ethnicity	Diagnostic criteria (year)	Ref.
Wang et al. 2016	80	3 h OGTT (75 g)	Total plasma GLP-1 ELISA, Westand Biological Technology	Not determined (ND)	No change in time point GLP-1levels, ΔGLP-1, or 3 h AUC GLP-1 versus NGT	ND	Matched	Matched	Han Chinese	WHO (‘98)	[7]

Faerch et al. 2015	1462	2 h OGTT (75 g)	Total GLP-1: (7–36)NH_2_ & (9–36)NH_2_ Antiserum 89390. RIA	Women: rAUC_0–30_: reduced versus NGT rAUC_0–120_: reduced versus NGT iAUC_120_: reduced versus NGT	Women: rAUC_0–30_: reduced versus NGT rAUC_0–120_: reduced versus NGT	Women: rAUC_0–120_: reduced versus NGT iAUC_30_: reduced versus NGT iAUC_120_: reduced versus NGT Both sexes: reduced 120 min versus NGT	Not matched	Not matched	Danish	WHO (‘06)	[9]

Yabe et al. 2015	102	2 h OGTT (75 g) MTT (480 kcal)	Intact: two-site sandwich ELISA GLP-1: (7–36)NH_2_ & (7–37) DPP-4 inhibitor: √ Total: GLP-1 (7–36)NH_2_ & (9–36)NH_2_ Antiserum 89390—Lab. of Holst JJ.	ND (fasting plasma glucose ≥110 mg/dl/6,1 mmol/l excluded)	No change in intact or total GLP-1 in fasting or post-prandial(GLP-1 levels and 2 h AUC) versus NGT in both challenges	ND	IGT and NGT matched	IGT and NGT not matched	Japanese	Japanese Diabetes Society	[19]

Zheng et al. 2014	474	OGTT (75 g)	Active GLP-1: (7–36)NH_2_ & (7–37) Active ELISA, Millipore	Fasting GLP-1 reduced in NGT subjects who developed prediabetes 4 year later Prediabetes = i-IFG, i-IGT, IFG/IGT—not distinguished between subtypes			Not matched. Higher in subjects developing prediabetes	Not matched. Higher in subjects developing prediabetes	Chinese	WHO (‘98)	[25]

Hussein et al. 2014	80	30 min OGTT (75 g)	Total GLP-1 ELISA (4141), DRG^®^ (Springfield, NJ)	No difference in 30 min GLP-1 in obese IFG versus obese NGT	ND	No difference in 30 min GLP-1 in obese IFG/IGT versus obese NGT	Normal weight versus obese (obese groups: matched)	Not matched	Egyptian	ADA (‘06)	[27]

Fernandez-Garcia et al. 2014	40	3 h high-fat meal (60 g)	Active GLP-1: (7–36)NH_2_ EIA, Phoenix Pharmaceuticals DPP-4-inhibitor: ?	No diff between 0 and 180 min GLP-1. Lower 180 min GLP-1 in IFG versus Low-Ins-Res-NGT	ND	ND	Morbidly obese. Matched	Matched	Spanish	ADA (‘11)	[17]

Shen et al. 2013	43	2 h OGTT (75 g)	Total GLP-1 RIA, Millipore	ND	No change in fasting GLP-1 versus NGT. GLP-1 levels at 30, 60, and 90 min and 120 min-AUCreduced versus NGT	ND	Matched	Matched	Chinese	WHO (‘99)	[28]

Zhang et al. 2012	531	2 h OGTT (75 g)	Total GLP-1 ELISA, USCNLIFE™ kits (Uscnlife Science & Technology Company)	No change: fasting GLP-1, 2 h GLP-1, and ΔGLP-1	No change: fasting GLP-1 and 2 h GLP-1. Reduced ΔGLP-1versus NGT	Reduced fasting GLP-1 versus i-IGT; reduced 2 h GLP-1 versus NGT, i-IFG, i-IGT; reduced ΔGLP-1 versus NGT, i-IFG, i-IGT	Matched	Matched	Han Chinese	ADA (‘06)	[29]

Smushkin et al. 2012	165	2 h OGTT (75 g)	Active GLP-1: N-terminusepitope + epitope in middle of peptide Inactive GLP-1: N-terminus epitope + epitope in middle of peptide Total: active + inactive DPP-4 inhibitor: √ Theranos System: fully automated, Chemiluminescence ELISA	No change in either active or total GLP-1 in fasting or postprandial response (max, 2 h AUC, 2 h AAB) versus NFG/NGT	No change in fasting active or total GLP-1. Increased integrated incremental concentrations (AAB) of total (2 h) GLP-1 versus NFG/NGT. Otherwise, no change in active or total postprandial GLP-1 response versus NFG/NGT	No change in either active or total GLP-1 in fasting or postprandial response (max, 2 h AUC, 2 h AAB) versus NFG/NGT	Not matched	Not matched	American	ADA (‘03≤)	[14]

Pala et al. 2010	56	2 h OGTT (75 g)	Active GLP-1: (7–36)NH_2_ & (7–37) ELISA, Linco—N-terminal specific DPP-4 inhibitor: √	ND	Decreased 30 min GLP-1 and 2 h AUC GLP-1 in IGT versus NGT	ND	Matched	Matched	Not reported	WHO (‘98)	[26]

Nathanson et al. 2010	509 men	60 min OGTT (75 g)	Total GLP-1: (7–36)NH_2_ & (9–36) RIA, C-terminal specific (7–36)NH_2_	ND	No change in fasting and 60 min GLP-1 versus NGT. Reduced ΔGLP-1 versus NGT	ND	Not reported	Matched	Swedish	WHO (‘99)	[30]

Lee et al. 2010	40	2 h OGTT (75 g) MTT(480 kcal)	Active GLP-1 ELISA, Linco DPP-4 inhibitor: √	ND	No change in peak GLP-1 or 2 h–iAUC in IGT versus NGT in both challenges	ND	Not matched	Matched	Japanese	WHO	[20]

Faerch et al. 2008	66	3 h OGTT (75 g) (IVGTT) (Clamp)	Total GLP-1: (7–36)NH_2_ & (9–36)NH_2_ Antiserum number 89390 Department of Biomedical Sciences—University of Copenhagen	Fasting GLP-1: no change versus NGT. Significantly higher 3 h AUC versus NGT	Fasting and 3 h AUC GLP-1: no change versus NGT	ND	Significant higher in i-IFG, i-IGT versus NGT	Matched	Europid	WHO (‘99)	[21]

Muscelli et al. 2008	51	3 h OGTT (75 g) (Isoglycaemic IV test)	Total GLP-1: 7-36NH_2_ & 9-36NH_2_ Antiserum number 89390. RIA	ND	No change in either GLP-1 levels or 3 h AUC GLP-1 versus NGT	ND	Matched	Matched	Not reported	ADA (‘97)	[22]

Laakso et al. 2008	278: GLP-1 (874)	2 h OGTT (75 g) (IVGTT) (Clamp)	Total GLP-1: (7–36)NH_2_ & (9–36)NH_2_ Antiserum number 89390	Reduced 15, 90, and 120 min GLP-1 versus NGT. Reduced AUC_0–240_ versus NGT	Reduced 15, 90, and 120 min GLP-1 versus NGT. Reduced AUC_0–240_ versus NGT	Reduced 15, 90, and 120 min GLP-1 versus NGT. Reduced AUC_0–240_ versus NGT	—	—	Danish	ADA (‘03)	[23]

Vollmer et al. 2008	48	4 h OGTT (75 g) Mixed meal (820 kcal)	Total GLP-1: (7–36)NH_2_ & (9–36)NH_2_ Antiserum number 89390. RIA	ND	No change in GLP-1levels in both challenges versus NGT	ND	Matched	Matched	Not reported	WHO	[24]

Rask et al. 2004	30 women	3 h OGTT (75 g)	Total GLP-1: (7–36)NH_2_ & (9–36)NH_2_ RIA	ND	No change in fasting GLP-1, GLP-1 levels, 30-minAUC, 60 min AUC GLP-1 versus NGT. Reduced first 30 min [GLP-1] increase versus NGT	ND	Matched	Matched	Caucasian	Not reported (Fulfil WHO & ADA)	[31]

Toft-Nielsen et al. 2001	102	4 h mixed meal (2250 kJ)	Total GLP-1: (7–36)NH_2_ & (9–36)NH_2_ Antiserum number 89390. RIA	ND	No change in fasting GLP-1 or 4 h AUC GLP-1 versus NGT	ND	Not matched	Matched	Danish	WHO (‘85)	[18]

Ahrén et al. 1997	13 women	2 h OGTT (75 g)	Total GLP-1: (7–36)NH_2_ & (9–36)NH_2_ Antiserum number 89390. RIA	ND	No change in fasting GLP-1, GLP-1 increase (AUC_0–60_) or GLP-1 decrease versus NGT	ND	Matched	Matched	Not reported	WHO (‘85)	[32]

## Abbreviations

IGT: Impaired glucose tolerance
IFG: Impaired fasting glucose
i-IGT: Isolated impaired glucose tolerance
i-IFG: Isolated impaired fasting glucose
rAUC: Relative area under the curve
iAUC: Incremental area under the curve
tAUC: Total area under the curve
GLUTag L cell model: A GLP-1-secreting cell line (source: glucagon-producing enteroendocrine cell tumor that arose in transgenic mice generated on an outbred CD-1 background [38]).
